# Th17 Effector Cells Support B Cell Responses Outside of Germinal Centres

**DOI:** 10.1371/journal.pone.0049715

**Published:** 2012-11-16

**Authors:** Agapitos Patakas, Robert A. Benson, David R. Withers, Paola Conigliaro, Iain B. McInnes, James M. Brewer, Paul Garside

**Affiliations:** 1 Institute of Immunology, Infection and Inflammation, Glasgow Biomedical Research Centre, University of Glasgow, Glasgow, United Kingdom; 2 MRC Centre for Immune Regulation, Institute for Biomedical Research, Birmingham Medical School, Birmingham, England, United Kingdom; 3 Unit of Rheumatology, Department of Internal Medicine, University of Rome Tor Vergata, Rome, Italy; University of Florence, Italy

## Abstract

Th17 cells are pro-inflammatory CD4^+^T cells, which are important in immune responses against fungal pathogens and extracellular bacteria and have also been implicated in various autoimmune syndromes. However, their role in supporting B cell responses in these scenarios remains unclear, representing a significant lapse in our understanding of the role Th17 play in vaccine responses and the regulation of autoimmunity. We employed T cell and B cell receptor transgenic mice specific for model antigens, and adoptive transfer approaches that allowed the tracking of cognate B and T cells *in situ* and *ex vivo* using immunological methods. We have found that T cells activated under Th17 polarising conditions have a greater capacity to provide cognate B cell help compared with Th1 polarised populations, supporting higher expansion of antigen specific B cells and enhanced antibody titres. This advantage is associated with the increased persistence of Th17 polarised cells in areas of the lymph nodes where they can provide help (i.e. the B cell follicles). Also the Th17 cells are characterised by their higher expression of ICOS, a costimulatory molecule important for B cell help. Surprisingly, contrary to published reports, Th17 cells were not detected inside germinal centres, although they were found in close proximity to cognate B cells in the follicle early in the genesis of the humoral immune response. These data indicate that, Th17 cells have a more significant role earlier in the initiation/development of the germinal centre response and/or germinal centre-independent events, consistent with their early effector status.

## Introduction

Th17 T cells are pro-inflammatory cells that produce cytokines such as IL-17A, IL-17F, IL-21 and IL-22 and are identified by the lineage transcription factor RORγt [1]. Both human and animal studies have demonstrated an important role for Th17 cells in mucosal and epithelial defence, especially against fungi and extracellular bacteria [1], [2]. Due to the production of cytokines such as IL-17 and IL-22, Th17 have been linked to various inflammatory syndromes namely psoriasis and Crohn’s disease [3]. Their role as effector cells has generally been investigated in the tissue site where they induce events such as neutrophil accumulation and production of antimicrobial peptides [1], [4], [5]. On the other hand, their function in the lymph node (LN), and specifically their ability to provide help to B cells, has not been analysed to the same extent. This is important as in several protective or pathogenic conditions Th17 cells appear to be involved in scenarios where antibodies play an important role [1].

During the development of a T cell-dependent antibody response, several distinct T-B cognate interactions take place in the secondary lymphoid organs [6]. These events are co-ordinated, take place in defined areas of secondary lymphoid organs and can result in the generation of either early memory B cells, germinal centre (GC) B cells or extra-follicular plasma cells [6]. It has been proposed that the T cells providing B cell help constitute a distinct Th subset, termed T follicular helper (TFH) [7]_ENREF_4. These cells are characterised by the high expression of the chemokine receptor CXCR5, co-stimulatory molecules such as ICOS and PD-1, the cytokine IL-21 and the transcription factor Bcl-6 [7]. However, there is also evidence to indicate that effector cells can also support B cell responses. Firstly, most activated CD4^+^cells up-regulate CXCR5 transiently, regardless of the immune response elicited [8]. In addition, we have previously demonstrated that *in vitro* and *in vivo* generated Th1 and Th2 cells can migrate to the B cell follicle where they support cognate B cells [9], [10], whereas cells with a TFH phenotype have been shown to express the Th2 transcription factor GATA-3 [11]. Specifically for Th17 cells, few studies have investigated their role in a humoral immune response [12], [13]. Studies in the BXD-2 mouse strain that develop a lupus like syndrome, demonstrate that IL-17 promotes the formation of, and stabilises, the developing GC. Interestingly, it has been demonstrated that IL-17-expressing CD4^+^T cells can be located in GCs in proximity to IL-17R^+^B cells [12]. This is controversial as other studies suggest that, unlike cytokines such as IL-4 and IL-21, IL-17 is not characteristic of GC T cells [14]. This might suggest that the results in the BXD-2 mice might not represent a physiological process but to reflect the autoimmune background of the strain.

In this study we attempted to investigate the role of Th17 cells in the development of a humoral immune response, in a manner that allowed us to track and manipulate the cognate B and T cell populations [9], [10]. Our group and others have demonstrated that this is a good model for analysis of cell localisation during the early phase of the B response and the GC reaction [9], [10], [15]–[17]. In addition, we compared the relative ability of Th17 and Th1 cells to support B cell responses, as these subsets appear to have overlapping functions in protective immunity and have been linked to autoimmune syndromes.

We demonstrate that cells polarised under Th17 conditions appear to be more efficient at supporting B cell responses compared to their Th1 counterparts, inducing higher expansion of cognate B cells, increased levels of class switched antibodies and greater numbers of GC B cells. There was also a difference in the character of the antibody response, with cells polarised under Th17 conditions promoting higher IgG1 levels whereas their Th1 counterparts supported higher IgG2c responses. We show that this relative advantage may be related to differences in the clonal expansion between the two populations, as cells polarised under Th17 conditions persisted longer and in higher numbers in the dLNs and B cell follicles, and expressed higher levels of the co-stimulatory molecule ICOS than their Th1 counterparts. Furthermore, Th17 cells were located in close proximity to cognate B cells at early time points in the immune response but were not found in the GC. This suggests that these cells are not involved in the events that take place in the GC but rather, they are crucial for the very early, extra-germinal centre antibody response that is beneficial in many infections.

## Materials and Methods

### Animals

Homozygous DO11.10 BALB/c (H-2^d/d^) mice, expressing the DO11.10 T cell receptor (TcR) specific for chicken ovalbumin (OVA) peptide 323–339/I-A^d^, were used as CD4^+^cell donors. Mice heterozygous for the anti-hen egg lysozyme (HEL) IgM^a^ and IgD^a^ transgenes on the BALB/c background (MD4) were screened by flow cytomentry and animals positive animals were used as donors of transgenic B cells [18]. IgH^b^ BALB/c (H-2^d/d^, IgM^b^) mice [19] were used as recipients of the transgenic B cells. All procedures were carried out according to UK Home Office regulations, under UK Home Office Project Licence PPL/603658, and approved by the Glasgow University animal use committee.

### T Cell Purification and Polarisations and Cell Culture

CD4^+^T cells were purified from pooled LNs and spleens from DO11.10 mice by negative selection (Miltenyi Biotec, Surrey, UK). Positive fractions were treated with mitomycin C (Sigma-Aldrich, Irvine, UK) and used as APCs. Th1 differentiation was induced by culturing CD4^+^cells with mitomycin C treated splenocytes as antigen presenting cells (APCs) in the presence of anti-IL-4 antibody (2 µg/ml, R&D Systems, Abingdon, UK), IL-12 (10 ng/ml, R&D Systems) and OVA_323–339_ (0.5 µg/ml, Cambidge Bioscience, Cambridge, UK). Th17 differentiation was induced by culturing CD4^+^T cells and APCs in the presence of anti-IL-4 (10 µg/ml), anti-IFNγ (10 µg/ml, BD Biosciences, Oxford, UK), IL-6 (20 ng/ml), TGFβ (1 ng/ml,), IL-23 (10 ng/ml), IL-1β (10 ng/ml) (R&D Systems) and OVA_323–339_ (0.5 µg/ml). In both Th1 and Th17 polarisation cells were cultured in IMDM with 10%FCS, 2 mM L-glutamine, 100 U/ml penicillin and 100 µg/ml streptomycin for 4 days, and were then harvested for transfer. Bone marrow dendritic cells (BMDCs) were generated from BALB/c mice in the presence of GM-CSF conditioned medium for seven days as previously described [20]. To assess the viability of the polarised transgenic T cells, Th1 or Th17 polarised T cells were co-cultured with LPS activated or not BMDCs for 24 or 48 hrs in the presence or absence of OVA. Viability was assessed using the Annexin-V FITC kit (Miltenyi Biotec). Carboxyfluorescein succinimidyl ester (CFSE) dilution assay was performed using CellTrace™ CFSE proliferation kit (Invitrogen) according to manufacturer’s instructions.

### Adoptive Transfers and Immunisations

Single cell suspensions of LNs and spleens were prepared from MD4 mice as described previously^5^. The proportion of transgenic B and T cells was determined by flow cytometry. 2×10^6^ transgenic B cells and 2×10^6^ transgenic Th1 or Th17 populations were adoptively transferred to age-matched IgHb BALB/c recipients. One day post transfer mice were immunised with 130 µg of OVA-HEL conjugate in CFA. Control mice were injected with PBS. Conjugated OVA-HEL was prepared as previously described [9].

### Flow Cytometry

At days 3, 7 and 10 post-challenge single cell suspensions were produced from LNs draining the site of immunisation from recipient mice and aliquots were stained with combinations of anti-CD4 PercP (RM4-5, BD Biosciences), KJ1.26 FITC (eBioscience, Hatfield, UK), biotinylated anti-CXCR5 (2G8, BD Biosciences), anti-ICOS PE or PE-Cy5 (7E.17G9, BD Biosciences), anti-B220 APC (RA3-6B2, eBioscience), anti-GL-7 FITC (GL7), anti-FAS PE (Jo2), PD-1 PE-Cy7 (RPM1-30, Biolegend) and biotinylated anti-IgMa(DS-1) (BD Biosciences). For intracellular cytokine staining cells were stimulated with PMA (50 ng/ml) and ionomycin (500 ng/ml) (Sigma-Aldrich) in the presence of Golgi Plug™ (BD biosciences) for 4 hrs and stained for IFNγ APC (XMG1.2) and IL-17 PE (TC11-18H10) (BD Biosciences) as described previously^6^.For nuclear staining the BD transcription factor buffer set was used according to manufacturer’s instructions. Anti-human/mouse RORγt APC (AFKJS-9, eBioscience), anti-Bcl-6 PE (K112-91, BD Pharmigen) and anti-Tbet PE-Cy7 conjugated antibodies (eBio4B10, eBioscience) were used to identify transcription factor expression. Appropriate isotype controls were used in all cases (BD Biosciences). Samples were analysed using FlowJo (Tree Star Inc, Ashland, OR, USA).

### ELISA

Anti-OVA and anti-HEL antibodies were detected by ELISA as previously described [9]. For detection of IgG2c antibodies a mouse anti-IgG2a antibody was used (Southern Biotech, AL, USA) that cross-react with IgG2c.

### Immunofluorescence

At days 3,7 and 10 post immunisation draining LNs were snap-frozen in OCT embedding medium (VWR) and stored at −80°C. 8 µm sections were cut and fixed in acetone. For detecting the transgenic T cells sections were stained with biotinylated KJ1.26 (Miltenyi Biotec) followed by steptavidin Alexa Fluor® 647 (Invitrogen, Paisley, UK). For identifying RORγt expressing cells, section were stained with anti-RORγt (eBioscience, AFKJS-9), followed with anti-Rat IgG FITC (Southern Biotech, Birmingham, Alabama, USA), rabbit anti-FITC Alexa Fluor® 488 (Invitrogen), anti-rabbit Alexa-Fluor® 488 (Invitrogen). B cell follicles were identified by either staining with anti-B220 FITC (RA3-6B2, eBioscience). Transgenic B cells were identified using a biotinylated anti-IgM^a^ (MA-69, Biolegend, Cambridge, UK) antibody as host B cells express IgM^b^ and streptavidin Alexa-Fluor® 546 (Invitrogen). GCs were identified with biotinylated-peanut agglutinin and streptavidin Brilliant Violet 421™ (Biolegend). Images were taken using LSM510 META Confocal Imaging System (Zeiss) and were analysed using Volocity® software (Perkin Elmer, Cambridge, UK). The tile scan function of the microscope allowed imaging of the full surface of the LN section. Areas of interest were drawn around the borders of the sections or around the follicular regions. This allowed the calculation of the surface of LN section and follicular area respectively. In addition the number of transgenic T cells in the section and in the follicular areas could be calculated (Fig S1). The localisation of T cells in the follicular area was calculated as a fraction of the proportion of KJ1.26^+^cells in the follicular area (Tg T cells_follicle_/Tg T cells_total_) to the proportion of the follicular surface (area_follicle_/area_total_) (Fig S1). This gave a number that was normalised for both T cell expansion (Tg T cells_total_) and follicular area (area_follicle_), and thus differences observed would be due to follicular localisation and not higher clonal expansion or larger follicular area in a specific section.

### Statistics

Data were analysed using the GraphPad Prism® software. To test if the means of two samples are different the Student’s t-test was used. To compare the means of two or more samples ANOVA was used. When the interaction of two independent variables was tested two-way ANOVA was employed. A value of P<0.05 was considered as significant.

## Results

### Cells Polarised Under Th17 Conditions Demonstrate Greater Ability in Supporting B Cell Responses Compared to Th1 Polarised Cells

To investigate the relative ability of Th1 and Th17 cells to support B cells, an adoptive transfer approach was employed that allowed the tracking of antigen-specific B and T cells. This is an adaptation of a previously described model in which the response of BCR transgenic cells depends on cognate help provided by antigen specific transgenic T cells [9], [10], [15]. In brief, OVA-specific T cells from DO11.10 mice were polarised under Th1 or Th17 polarising conditions and adoptively transferred with hen egg lysozyme (HEL)-specific B cells from MD4 mice to IgH^b^ congenic recipient mice prior to HEL-OVA/CFA challenge (Fig S2A and C).

**Figure 1:**
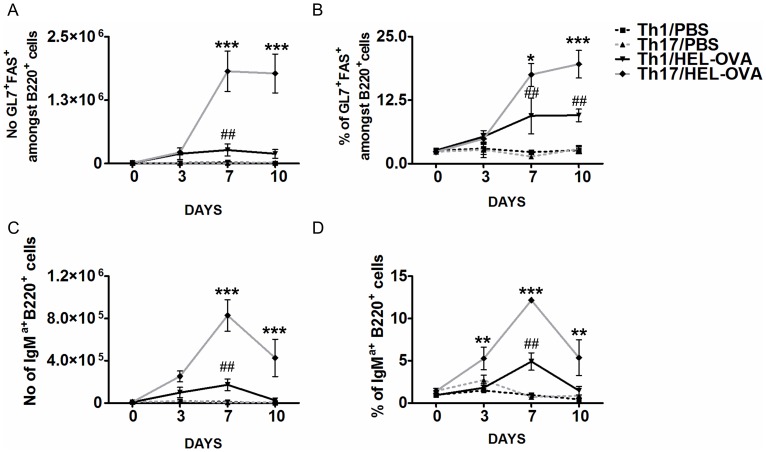
Cells polarised under Th17 conditions have a relative advantage in inducing antigen specific B cells responses compared to the Th1 population. The number (A) and percentage (B) of GC B cells in dLNs was assessed by flow cytometry 3, 7 and 10 days post-immunisation. GC B cells were considered B220^+^cells co-expressing GL-7 and FAS. In addition the number (C) and proportion (B) of HEL-specific transgenic B cells in dLNs was analysed by flow cytometry, recognised as B220^+^IgM^a+^. Results from PBS injected mice from each time point were averaged and presented as day 0. *: Th1/HEL-OVA vs. Th17/HEL-OVA, # Th1/PBS vs. Th1/HEL-OVA. Data represent mean ±SEM.#,*p<0.05, ##,**p<0.01, ###,***p<0.001 (n = 3). Similar results were obtained in one additional experiment.

The GC is known to be associated with T cell dependent antibody responses and is the site where clonal selection and expansion, class switching, somatic hypermutation and affinity maturation occur [21]. Based on this, the ability of cells polarised under Th1 or Th17 conditions to support the generation of GC B cells was initially investigated. GC B cells were identified by the expression of GL-7 and FAS by flow cytometry as done previously by ourselves and others (Fig S3B) [22], [23]. In both Th1 and Th17 recipient mice GC B cells were present at a higher percentage and number in the draining LN (dLN) than unimmunised controls by day 7 after immunisation (Fig. 1A and B). The number and percentage of GC B cells was significantly higher in mice that had received cells polarised under Th17 conditions compared to those that had received Th1 polarised cells (Fig. 1A and B). This was apparent both at days 7 and 10. To determine their ability to provide B cell help, we analysed the impact of Th1 and Th17 cells on the clonal expansion of cognate transgenic B cells, identified by their expression of IgM^a^ (Fig S3A). In the absence of immunisation, as anticipated, transgenic B cells did not expand in either Th1 or Th17 recipient mice. In response to HEL-OVA/CFA, mice that received the Th17 polarised population exhibited significantly higher antigen specific B cell clonal expansion compared with Th1 recipient mice (Fig. 1C and D). In both Th1 and Th17 recipient mice, the proportion and number of transgenic B cells was higher than in unimmunised groups (Fig. 1C and D) and clonal expansion peaked between days 3 and 7. However, in the case of Th17 recipients, B cell numbers had not returned to unimmunised levels by day 10 and it is likely that their number declines at a later time point (Fig. 1C and D). These data indicate an enhanced and sustained ability of cells polarised under Th17 conditions to support B cell clonal expansion compared with their Th1 counterparts.

**Figure 2:**
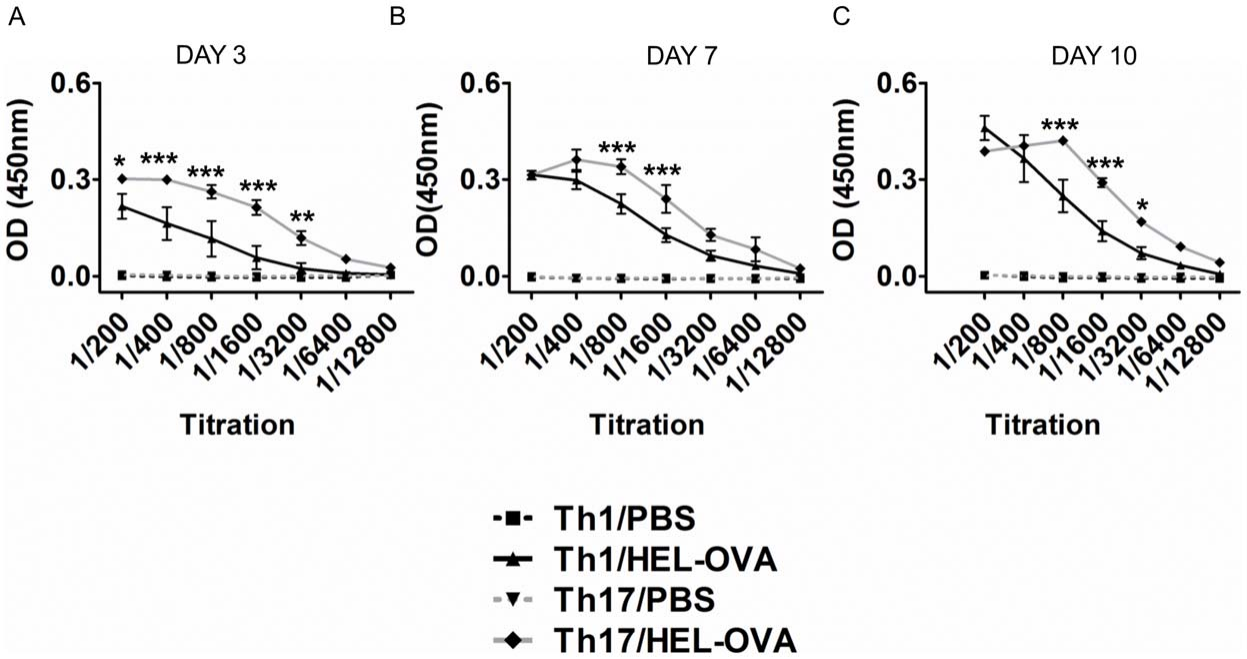
Th17 population induces higher titres of anti-HEL antibodies. Serum was taken from the animals and was assessed for the presence of HEL-specific IgM^a^ antibodies at day 3(A), 7(B) and 10(C). *: Th1/HEL-OVA vs. Th17/HEL-OVA. Data represent mean ±SEM. *p<0.05, **p<0.01, ***p<0.001 (n = 3). Similar results were obtained in one additional experiment.

To determine the functional status of the transgenic B cells, their ability to produce HEL specific antibodies was assessed by ELISA. Serum was sampled on days 3, 7 and 10 post immunisation and assessed for anti-HEL IgM^a^ antibody titres. T cells polarised under either Th1 or Th17 conditions could support antibody production, however the Th17 population promoted higher levels of HEL-specific antibodies compared with their Th1 counterparts, at all time-points investigated (Fig. 2A–C). In the absence of immunisation there was no antibody production in either Th1 or Th17 recipient mice. These results demonstrate that cells polarised under Th17 conditions have a relative advantage in supporting antigen specific antibody production.

**Figure 3:**
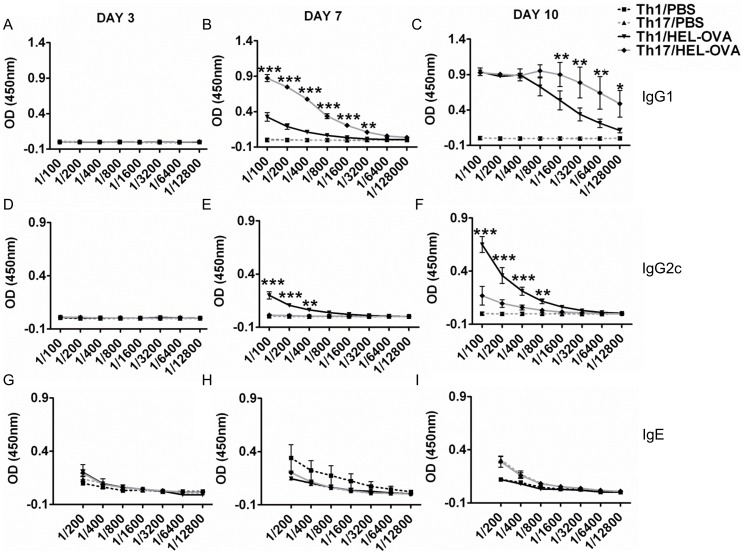
The antibody profile induced by the Th1 and Th17 population is different. As MD4 B cells does not class switch the IgG profile of the OVA specific response was assessed by ELISA. Serum was analysed for the presence of IgG1 (A–C) and IgG2c (D–F) anti-OVA antibodies at days 3, 7 and 10. Furthermore the levels of total IgE levels in the serum of the recipient mice were assessed at the same time points (G–I). *: Th1/HEL-OVA vs. Th17/HEL-OVA. Data represent mean ±SEM.*p<0.05, **p<0.01, ***p<0.001 (n = 3). Similar results were obtained in one additional experiment.

In addition to the response of the transferred transgenic B cells, the production of antibodies against OVA by endogenous host B cells allowed the evaluation of the relative ability of Th1 and Th17 populations to support isotype switching. Serum samples taken from mice at days 3, 7 and 10 post immunisation were analysed for the presence of OVA specific IgG1 (Fig. 3A–C), IgG2c (Fig. 3D–F) and total IgE levels (Fig. 3G–I). IgE antibodies were not detectable in either Th1 or Th17 recipient mice immunised with OVA-HEL/CFA (Fig. 3G–I). Prominent differences were noted in the OVA specific IgG response. Animals that have received cells polarised under Th17 conditions exhibited significantly higher OVA specific IgG1 titres from as early as day 7 compared with animals receiving cells polarised under Th1 conditions (Fig. 3E). This difference was still evident at day 10 (Fig. 3F). By contrast, recipients of the Th1 polarised cells displayed significantly higher titres of anti-OVA-IgG2c antibodies in response to immunisation compared with recipients of Th17 polarised cells. This was observed from day 7 and was still evident at the last time point investigated (day 10; Fig. 3G–I). Only at day 10 could anti-OVA IgG2c antibodies be detected in Th17 recipients (Fig. 3I), albeit at levels much lower than Th1 recipients. We also investigated endogenous anti-OVA-IgM^b^ antibodies, which did not differ between groups at the time points investigated (data not shown). These results show that T cells polarised under Th17 conditions promoted a predominantly IgG1 response, whereas the Th1 polarised population induced high IgG2c antibody levels.

### Cells Polarised Under Th17 Cells Persist Longer and in Higher Numbers in the Draining LNs Compared to their Th1 Counterparts

**Figure 4:**
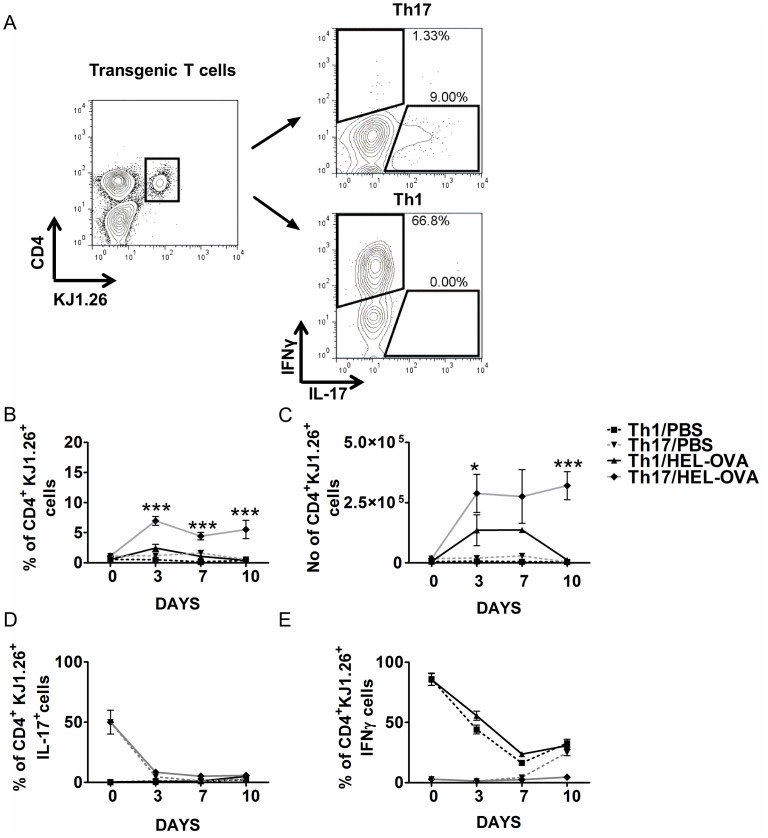
Differential expansion of Th1 and Th17 populations after adoptive transfer. Th1 or Th17 recipient mice were immunised with HEL-OVA in CFA or PBS and the expansion of transgenic T cells and their ability to produce IL-17 and IFNγ was analysed by FACS. Representative FACS plots demonstrating gating strategy are demonstrated (A). Collective data demonstrating the percentage (B) and number of (C) of transgenic T cells at days 3, 7 and 10 post-immunisation amongst draining lymph node cells was assessed by flow cytometry, based on the expression of CD4 and the clonotypic TcR recognised by the KJ1.26 antibody (A). Unimmunised controls from each time point were averaged and represented as day 0. At the same days cells from the dLNs were stimulated with PMA and ionomycin and the expression of IL-17 (D) and IFNγ (E) by the transferred transgenic T cells was assessed by flow cytometry. Day 0 represents the proportion of IL-17 or IFNγ population in the transferred polarised populations. Grey line represents Th17 immunised recipients, grey dotted line unimmunised Th17 recipients, black line Th1 immunised recipients and black dotted line Th1 unimmunised recipients. Data represent mean ±SEM. *: Th1/HEL-OVA vs. Th17/HEL-OVA, *p<0.05, **p<0.01, ***p<0.001 (n = 3). Similar results were obtained in two additional experiments.

The differences in the magnitude of antibody responses supported by the two T cell populations could have been attributed to differences in their *in vivo* expansion. To investigate this, dLNs were removed from the recipients at days 3, 7 and 10 post-immunisation and the transgenic T cells populations analysed by flow cytometry (Fig. 4A). As expected, in unimmunised mice that had received either Th1 or Th17 populations there was no transgenic T cell expansion, and cells were almost undetectable by day 10 (Fig. 4B and C) after transfer. Immunisation induced expansion of both populations, with greater levels being observed by cells polarised under Th17 conditions (Fig. 4B and C). Cells polarised under Th1 conditions accumulated in the dLNs, where their numbers peaked between day 3 and day 7, and reduced to levels of unimmunised mice by day 10. By contrast, cells polarised under Th17 conditions accumulated in the dLNs and persisted at this site in high numbers even up to the last time point investigated (day 10) (Fig. 4B and C). The persistence of Th17 compared with Th1 cells was also evident in spleen (Fig S4A and B) and therefore not restricted to dLN.

A characteristic of *in vitro* polarised Th17 cells is their phenotypic instability [24], [25]. Therefore, to determine the phenotype of the transferred populations, cells from the dLNs were stimulated with PMA and ionomycin, to induce synchronous cytokine production and the ability of the transgenic T cells to produce IL-17 or/and IFNγ was assessed by flow cytometry (Fig. 4D and E). In immunised and unimmunised animals, the transferred Th1 population exhibited a reduction in its ability to produce IFNγ (Fig. 4E), with no increase IL-17 production (Fig. 4D). The Th17 transferred population in both immunised and unimmunised mice experienced a much sharper decrease in the percentage of IL-17^+^transgenic T cells, with an 83% reduction by day 3 (Fig. 4C). The proportion of IFNγ-producing transgenic T cells was not affected by the transfer and remained relatively low (Fig. 4E). Interestingly, in unimmunised Th17 recipient mice the percentage of IFNγ producing tg T cells increased by day 10 to similar levels as Th1 recipients, however this was not evident in the immunised mice (4D). Previous studies have suggested that Th17 cells can transform into Th1 effector cells [24], however our data suggest this does not occur using the polarisation and immunisation schedule described here.

We also investigated by flow cytometry the expression of the signature transcription factors for Th17 and Th1 cells, T-bet and RORγt, in the transferred populations seven days post-immunisation (Fig S4C). This analysis, confirmed maintenance of the phenotype of the transferred populations, with cells polarised under Th1 conditions expressing higher levels of T-bet, whereas their Th17 counterparts expressed higher levels of RORγt.

### Cells Polarised Under Th17 Conditions Display Enhanced Viability Compared with their Th1 Counterparts

**Figure 5:**
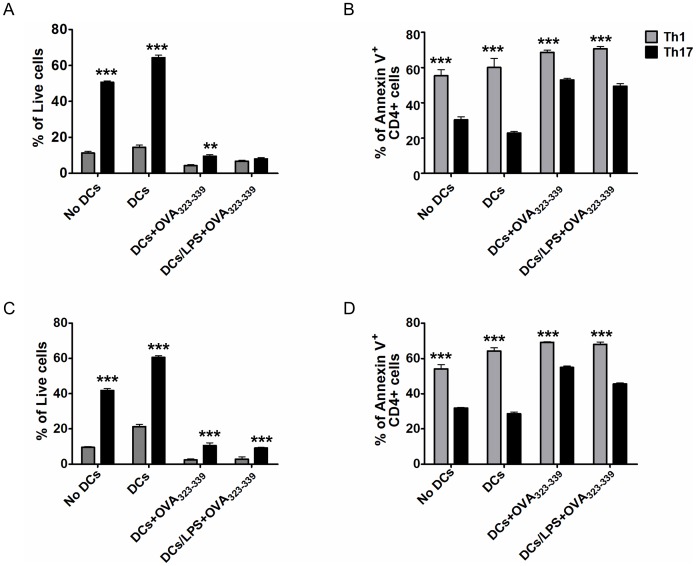
Cells polarised under Th17 conditions are more viable than their Th1 counterparts. MACS sorted CD4^+^T cells from DO11.10 mice were first polarised towards a Th1 (grey bars) or Th17 (black bars) phenotype and then cultured in the absence of any stimulus or with bone marrow-derived DCs, unpulsed or pulsed with OVA_323–339_, or OVA_323–339_ and LPS. Viability of transgenic and apoptosis levels of T cells was assessed at two time-points, 24 hrs (A–B) or 48 hrs (C–D) by PI and annexin V staining by flow cytometry. Lymphocytes were identified based on the FSC and SSC profile and transgenic T cells based on KJ1.26 staining and CD4 expression. Live cells were considered as annexin V and PI negative, whereas apoptotic cells were considered as annexin V positive. Data represent mean ±SEM.*p<0.05, **p<0.01, ***p<0.001 (n = 3).

The greater expansion and persistence of Th17 polarised cells may be explained by enhanced viability. The viability of Th1 or Th17 polarised transgenic cells was investigated upon restimulation with antigen pulsed and/or LPS activated bone marrow DC after 24 and 48 hours (Fig. 5A and C respectively). In the absence of antigen, cells polarised under Th17 conditions displayed enhanced viability by annexin V/PI staining compared with their Th1 counterparts. Similarly, in the presence of antigen, cells polarised under Th17 conditions displayed greater viability compared with the Th1 population, especially at 48 hrs (Fig. 5C). In both Th1 and Th17 populations the presence of antigen resulted in a significant reduction in the percentage of viable cells, suggesting the decrease in viability may be due to activation-induced cell death (AICD) (Fig. 5A and C). The increased survival of Th17 polarised cells is in agreement with a reduced rate of apoptosis compared with Th1 polarised cells as revealed by annexin V staining, a phenomenon evident in all conditions and time-points investigated (Fig. 5B and D). Furthermore, CFSE-dilution data (Fig S5) demonstrate that there is no difference in proliferation between the two populations (% of cells that diluted CFSE after OVA_323–339_ restimulation: Th1 27%, Th17 23%), which suggests that differences are due to viability and not to increased proliferation of the Th17 population. These data demonstrate that cells polarised under Th17 conditions are more viable than Th1 polarised cells.

### Th17 Polarised Population Expresses Higher Levels of ICOS Compared to the Th1 Population

**Figure 6:**
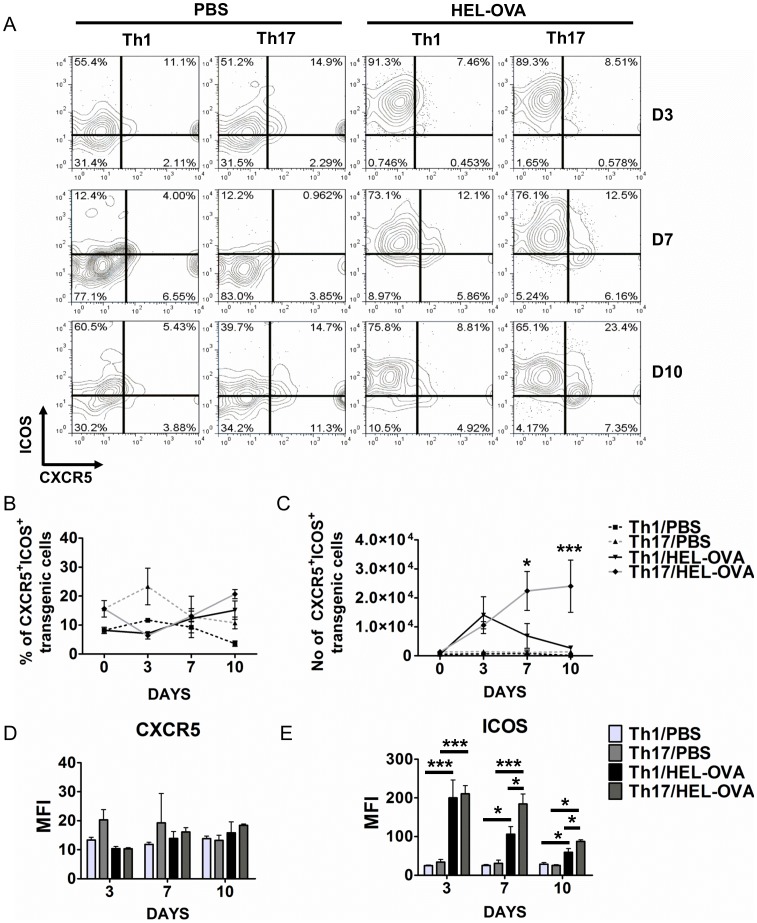
Cells polarised under Th17 conditions have a relative advantage in acquiring a follicular helper phenotype compared to their Th1 counterparts. The ability of transgenic T cells to acquire a potential to migrate into the B cell follicle and support B cell responses was analysed by the expression of CXCR5 and ICOS by flow cytometry at days 3, 7 and 10 post-immunisation (A). Lymphocytes were identified based on the FSC and SSC profile and transgenic T cells based on KJ1.26 staining and CD4 expression. The percentage (B) and number (C) of transgenic T cells that co-express ICOS and CXCR5 at days 3,7 and 10 are presented. Results from PBS injected mice from each time point were averaged and presented as day 0. D and E) Mean fluorescent intensity of CXCR5 (D) and ICOS (E) expressed on CD4^+^KJ1.26^+^transgenic T cell populations. Data represent mean ±SEM.*p<0.05, **p<0.01, ***p<0.001 (n = 3). Similar results were obtained in one additional experiment.

An important requirement of T cell-dependent B cell responses is the migration of activated antigen-specific T cell to the follicular region to allow interactions with cognate B cells. It has been reported that this is mediated by the down-regulation of the chemokine receptor CCR7 and concomitant up-regulation of CXCR5. In order to assess follicular migration and provision of co-stimulatory signals by the transferred Th1 and Th17 polarised populations, the expression of CXCR5 and ICOS was investigated at days 3, 7 and 10 post-immunisation (Fig. 6A–C). The proportion of ICOS^+^CXCR5^+^transgenic T cells was similar in both immunised and unimmunised, Th1 or Th17 recipients (Fig. 6B). This indicated that cells activated under either a Th1 or Th17 polarising environment are conditioned for follicular migration. Due to differences in clonal expansion, the number of transgenic ICOS^+^CXCR5^+^T cells was significantly higher in immunised mice (Fig. 6B and C). Notably, in the Th1 recipients the number of transgenic T cells that co-expressed ICOS and CXCR5 peaked at day 3 and declined to unimmunised levels by day 10. On the other hand, in Th17 recipient mice the number of transgenic cells with a TFH phenotype increased throughout the experiment and was significantly higher than in the Th1 population at both days 7 and 10 (Fig. 6C). In order to quantify the levels of expression of CXCR5 and ICOS by the transgenic T cells the mean fluorescence intensity (MFI) for these markers was calculated. Transgenic T cells in unimmunised mice expressed significantly lower levels of ICOS compared with their immunised counterparts at all-time points (Fig. 6D and E). Cells polarised under Th17 conditions expressed significantly higher levels of ICOS compared with Th1 polarised cells at days 7 and 10, suggesting a greater capacity to provide costimulatory help at these time points (Fig. 6E).

While CXCR5 and ICOS have been used extensively as markers of TFH cells, recent reports [26] suggest that differential levels of expression of CXCR5 and PD-1 can subdivide this population into TFH and central memory precursors, with co-expression of PD-1 and high levels of CXCR5 defining TFH cells. Thus we investigated the presence of transgenic T cells with this phenotype 7 days post-immunisation. Using flow cytometry, we could detect CXCR5^high^ PD-1^+^transgenic T cells in the dLNs in all immunised recipients, however mice that received Th17 cells had a higher percentage of TFH-like transgenic T cells compared with their Th1 counterparts (Fig S6). We also investigated the expression of the TFH transcription factor Bcl-6 by flow cytometry. In accordance with our previous data, cells polarised under Th17 conditions expressed the highest percentage of Bcl-6 compared with Th1 polarised cells seven days post-challenge (Fig S6). Collectively, this data suggest that cells polarised under Th17 conditions have a greater potential to acquire a phenotype that would support B cell responses.

### Cells Polarised Under Th17 Conditions Persist Longer in the B Cell Follicle Compared with the Th1 Population

**Figure 7:**
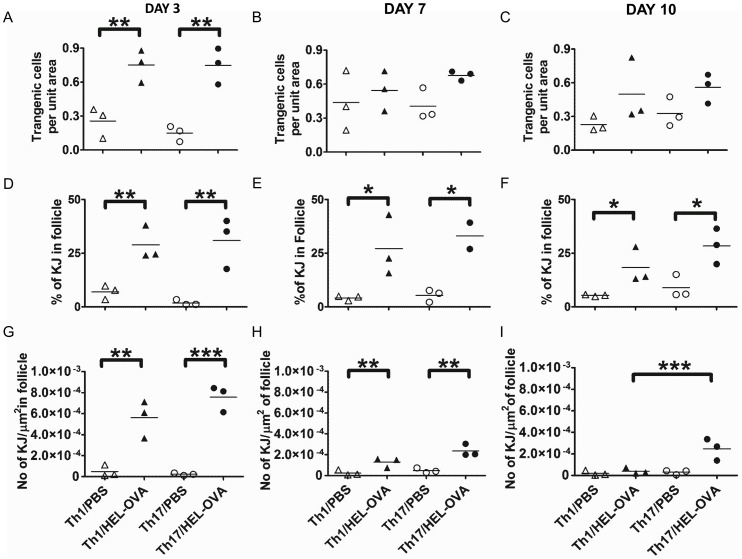
Relative ability of Th1 and Th17 populations to localise in the follicle. On days 3 (A), 7(B) and 10 (C) post-immunisation, the relative ability of the transgenic T cells to localise in the follicular area was quantified in situ by immunofluorescence staining using the Volocity® software as described in materials and methods and Fig S1. The percentage of the total transgenic T cells of the section that reside in the follicle (D–F) and the number of transgenic T cells per unit area of follicle (G–H) in the same time points are also presented. At least three sections were analysed from each animal and each point represents the mean of that. *p<0.05, **p<0.01, ***p<0.001 (n = 3). Similar results were obtained in one additional experiment.

As T cell migration to the follicle is required for provision of B cell help we investigated the anatomical localisation of Th1 and Th17 transgenic T cells in the LN at days 3, 7 and 10 post-immunisation (Fig S1). There were no differences between the Th1 and Th17 populations in respect to follicular localisation (Fig. 7A–C). On day 3, both Th1 and Th17 populations increased their follicular localisation in response to immunisation (Fig. 7A), suggesting that recruitment to the follicular area occurs rapidly and differences observed after that point are due to the greater number of antigen specific T cells in the dLNs of immunised mice (Fig. 7A–C). On the other hand, the percentage of the total transgenic T cells per section that resided in the follicle was higher in the immunised mice compared to the unimmunised controls at all-time points investigated (Fig. 7D–F). Furthermore, the number of transgenic cells per unit of follicular area was significantly increased in immunised groups (Fig. 7G–I). Importantly, in Th17 recipients this was observed even at day 10, unlike Th1 recipients, which were at unimmunised levels by this time point (Fig. 7I). This suggests that cells polarised under Th17 conditions persist in the follicular area for longer compared with their Th1 counterparts.

### Th17 Cells are Absent from Germinal Centres

**Figure 8:**
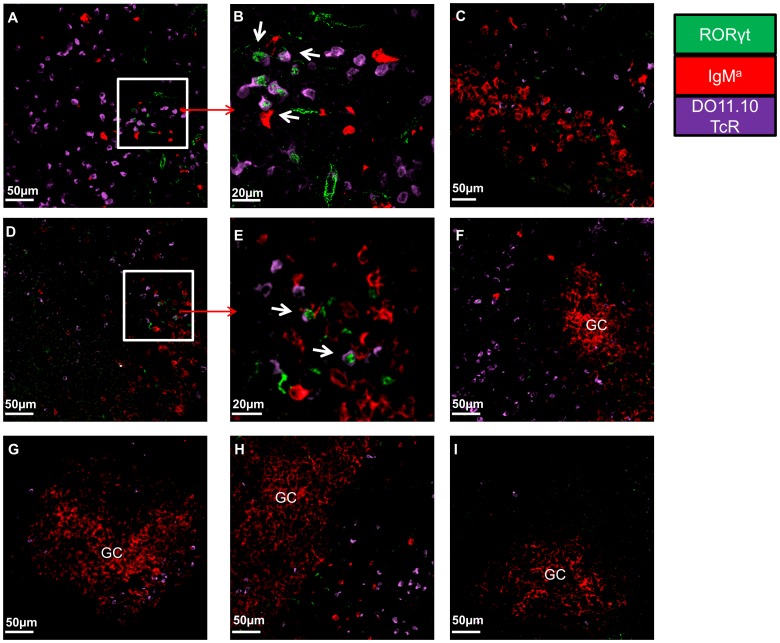
Th17 cells interact with cognate B cells. At days 3, 7 and 10 post immunisation dLNs were snapped frozen and were analysed for the presence of transgenic Th17 cells by the co-expression of the clonotypic DO11.10 TcR (PURPLE) and RORγt (GREEN). In addition transgenic B cells were detected by staining against IgM^a^ (RED). Representative pictures of dLN sections of: A and B) Th17 recipients 3 days post immunisation C) Th1 recipients 3 days post immunisation D and E) Th17 recipients 7 days post immunisation F) GC-like structures in Th17 recipient 7 days post immunisation G) GC-like structures in Th1 recipient 7 days post immunisation H) GC-like structures in Th17 recipient 10 days post immunisation I) GC-like structure in Th1 recipient 10 days post immunisation. Microscope: Carl Zeiss LSM510 META Confocal, Objectives: B) Zeiss planapochromat 40×/1 NA water objective; A, C–I) Zeiss PH 25×/0.85 NA water objective. Images were acquired using Zeiss LSM510 operating software and off-line image analysis (contrast enhancement and noise removal) were performed using Volocity® software. At least 3 random sections were imaged per mice and at least 4 pictures were acquired per section. Similar results were obtained in one additional experiment.

**Figure 9:**
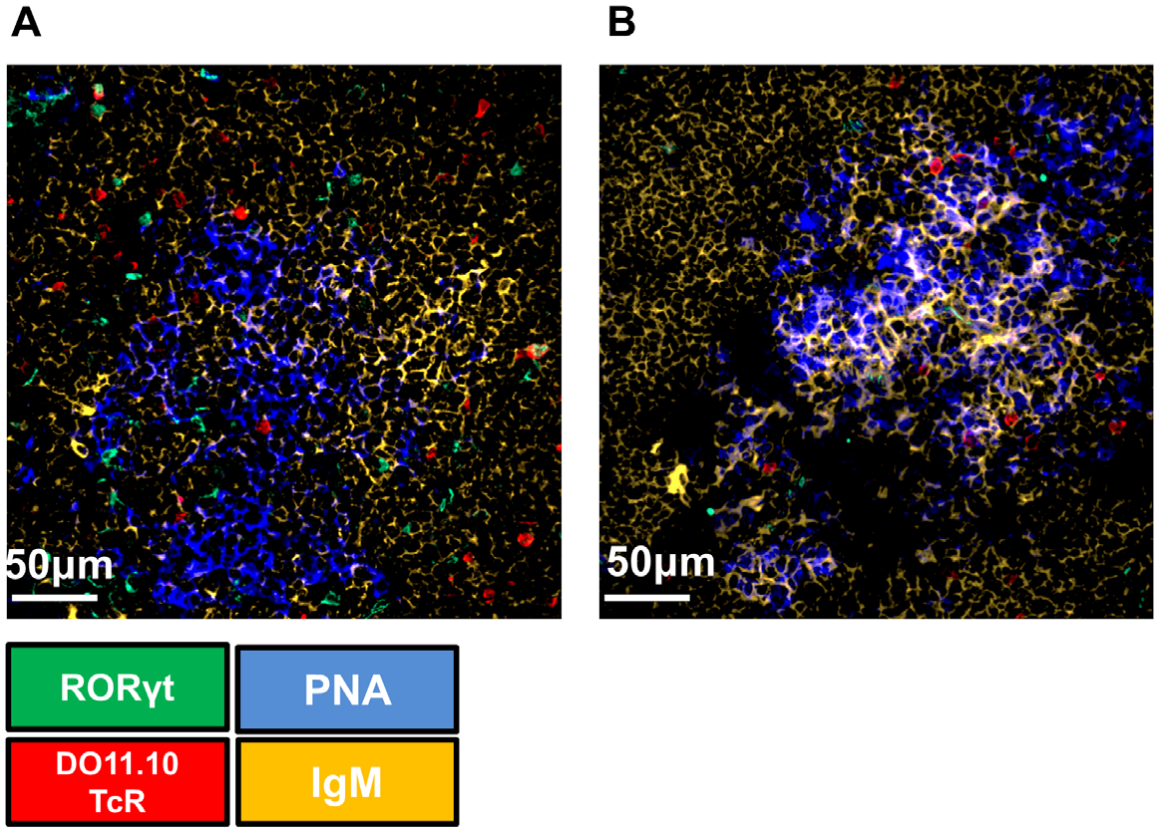
Antigen-specific Th17 cells cannot be located in the germinal centres. At days 7 (A) and 10 (B) post immunisation dLNs of Th17 recipient mice were snap frozen and were stained for PNA (BLUE), IgM (YELLOW), RORγ (GREEN), DO11.10 TcR (RED) to identify germinal centre, B cell follicles, and Th17 trasgenic T cells respectively. Microscope: Carl Zeiss LSM510 META Confocal, Objectives: Zeiss PH 40×/1.3NA air objective. Images were acquired using Zeiss LSM510 operating software and off-line image analysis (contrast enhancement and noise removal) was performed using Volocity® software. Three random sections per animal were used and at least 3 pictures per section were taken. Similar results were obtained in one additional experiment.

The location of the T-B cell interaction is an important factor in determining immunological outcome. For example, early T-B cell interactions in the follicular border have been linked to low affinity antibody producing plasmablasts [27], whereas interactions in the GC are more important for the differentiation of B cells into high affinity plasma cells and memory cells [28]. Therefore, we analysed *in situ* the expression of the Th17 specific transcription factor RORγ to determine the location of Th17 cells in relation to their cognate B cells as the humoral immune response developed. RORγ transgenic T cells could be detected only in Th17 recipients at each time point investigated (Fig. 8 and data not shown). Transgenic B cells could be detected in both Th1 and Th17 recipients. After day 7 the transgenic B cells formed large aggregates typical of GC. RORγ-expressing transgenic T cells could be detected in close proximity to cognate B cells at day 3 and 7 post immunisation, but not at day 10 (Fig. 8A, B, D and E). Interestingly, this could only be detected early in the developing humoral immune response and outside the GC-like structures (Fig. 8F–I). Even though transgenic T cells could be detected in these clusters, these never expressed RORγ (Fig. 8F–I). We confirmed that Th17 cells do not reside in GC by staining for them using peanut agglutinin (PNA). Transgenic Th17 cells could be detected around GCs, but not in them (Fig. 9).This suggests that Th17 are probably more important early in the follicular events that precede the GC reaction or for events that do not require GCs.

## Discussion

Currently, Th17 cells are considered to be pro-inflammatory effectors that cause tissue inflammation through the production of cytokines [29]–[32], with few studies investigating their role in humoral immune responses [12], [13]. Evaluation of the ability of Th17 cells to support B cell responses is important as in many of the conditions in which Th17 cells are involved, antibodies also play an important role [33]. Thus understanding and modulating Th17 function could be used to promote protective humoral immunity or alternatively to regulate autoimmune antibody production. In the current study, we have demonstrated that cells polarised under Th17 conditions have a relative advantage, compared with Th1 polarised cells, in providing cognate B cell help, resulting in greater antibody production. Furthermore, the two cell populations induced the production of different antibody isotypes by B cells, with the Th17 population inducing mainly IgG1, whereas the Th1 population induced higher titres of IgG2c. The greater ability of the Th17 population to support antibody production could be attributed to their higher clonal expansion, persistence in the B cell follicle and/or expression of ICOS compared with their Th1 counterparts. More importantly, in draining lymph nodes we could identify Th17 cells in close proximity to antigen specific B cells soon after antigen challenge, indicating cognate recognition. However, later in the response Th17 cells were absent from GCs, suggesting that other Th cells support this phase of the response. These data collectively demonstrate that Th17 polarised populations can induce a quantitatively and qualitatively distinct humoral immune response from that supported by cells polarised under Th1 conditions, which could be potentially useful for the development of vaccines against pathogens such as fungi.

The greater ability of cells polarised under Th17 conditions, compared with their Th1 counterparts in supporting B cell responses is evident through their greater induction of GC B cells, higher expansion of cognate B cells and antibody production by these cells. The difference between the two populations is not limited to the magnitude of antibody response but also extends to its character. In mice that received cells polarised under Th17 conditions, the IgG response was characterized by the IgG1 isotype and very low levels of IgG2c, whereas Th1 recipients by IgG2c. The IgG2c (equivalent of IgG2a in BALB/c mice) profile of the Th1 response is not a surprise as the role of IFNγ in IgG2a class switching is well established [9], [10], [34]. Our findings agree with a recent report which demonstrated that the antibody class profile induced by Th17 polarised populations is characterised mainly by IgG1, secondly by IgG2b and low levels of IgG2a antibodies [13]. The absence of IgE antibodies from the Th17 population recipients suggests Th2 cells are not involved in the humoral response and also fits with a proposed regulatory role for Th17 cells in IgE production as shown in patients with Job’s syndrome that have reduced Th17 responses and are characterised by hyper-IgE [35]. The difference in the antibody profile induced by the two populations could reflect their ability to provide protective immunity against different types of pathogens. For example, the lower levels of IgG2c antibodies in the Th17 recipients may suggest reduced involvement of complement mediated clearance of pathogens, compared to Th1 recipients. This, however, needs to be confirmed using *in vivo* models of infection that preferentially induce or require Th1 or Th17 responses for resolution.

One possible explanation for the greater ability of cells polarised under Th17 conditions to support B cell responses is their greater clonal expansion and persistence in the dLN compared with their Th1 counterparts. This could be attributed to the greater viability of the Th17 population. Indeed, reports suggest that Th17 cells are more resistant to AICD than Th1 cells, a phenomenon possibly mediated by a reduced expression of FasL by Th17 polarised populations [36]. Furthermore, there is a documented role for IFNγ in driving AICD of effector T cells [37], [38]_ENREF_31, suggesting that this cytokine could mediate increased cell death in the Th1 polarised cells. However, differential distribution of the two populations in the tissues could also account for the observed differences. A recent study suggests that Th17 polarised cells are not as efficient as Th1 cells at migrating to non-lymphoid tissues, due to a reduced expression of the chemokine receptors CCR5 and CXCR3 [39]. This difference in the number of transgenic T cells in the LN could have profound effects in the humoral immune response. Indeed, dynamic imaging studies using multiphoton microscopy and antigen targeting to B cells revealed that T cell help is the limiting factor for GC intrazonal migration and B cell clonal expansion [40], [41]. It is therefore possible that the higher numbers of transgenic T cells in the lymphoid compartment of Th17 recipient mice increases the availability of T cell help to B cells both before and after the formation of the GC resulting in more robust antibody responses. Apart from the higher numbers, we demonstrate that the cells polarised under Th17 conditions also expresses higher ICOS levels compared with the Th1 polarised population. ICOS is a CD28-like molecule which is crucial for T cell dependent antibody responses and is highly expressed by TFH cells [42], [43]. Absence of this molecule leads to severe defects in the GC reaction, whereas over-expression is linked to autoimmune syndromes such as SLE [44]–[47]. It is therefore possible that a combination of high number and a higher ability to provide costimulation leads to a more robust B cells response in Th17 population recipients.

The spatiotemporal characteristics of the T-B cell interaction are known to influence the outcome of the B cell response. For example, early interactions in the T-B cell border could lead to the development of GCs or early plasma-blasts and memory B cells that have not undergone class switching [6]. Interaction in the GC will lead to isotype switching, affinity maturation and generation of memory B cells [6]. We demonstrated, for the first time to our knowledge, RORγ expressing Th17 cells localising in close proximity to cognate B cells. However, this was evident only early in the developing response and never in GCs, suggesting that Th17 cells participate only in extra-germinal centre B cell responses. Our data contradict previous reports in BXD2 mice in which IL-17 producing CD4 T cells were identified in germinal centres [12]. This data however may represent a pathological phenomenon related to autoimmune phenotype of the mouse strain and not a normal humoral immune response. As in our studies we did not use purified Th17 T cells, we cannot discriminate the relative importance of Th17 vs. non-Th17 cells in the provision of B cell help. This would require Th17 reporter mice, such as the RORγ-EGFP mice, that would allow both intra-vital imaging of the Th17-B cell cognate interaction and fate mapping of the antigen-specific T cells as the GC reaction develops. Indeed, from our studies we cannot exclude that Th17 cells interacting with cognate B cells do not eventually down-regulate RORγt and express markers such as Bcl-6, becoming TFH cells. Indeed, our data demonstrate that cells polarised under Th17 conditions upregulate TFH markers such as Bcl-6, ICOS, PD-1 and CXCR5 to a greater extent than the Th1 population (Fig S6). However, it remains unclear whether this is due to transformation of Th17 effector cells to TFH or to their *de novo* generation from unpolarised cells. Recent reports have suggested that the presence of IL-6 and IL-21 can lead to *in vitro* generated TFH cells [48]. It therefore possible that IL-6 present in the Th17 cultures, conditions cells towards a TFH-like phenotype. In fact, a high percentage of the Th17 cells produced IL-21, a characteristic cytokine for both Th17 and TFH cells.

On the other hand, our data suggest that Th17 effector cells could co-ordinate early B cell responses, such as the formation of early plasma blasts until the germinal centre reaction is established, which is probably coordinated by other Th subtypes like the Bcl-6 expressing TFH cells.

In conclusion, we demonstrate that cells polarised under Th17 conditions induce a quantitatively and qualitatively different humoral immune response compared with Th1 cells, a phenomenon that should be taken into consideration especially in adjuvant development for pathogens that have been linked to Th17 cells, such as fungi.

## Supporting Information

Figure S1 **Analysis of localisation of transgenic T cells in the dLNs.** A) Tile scan images of dLN sections acquired by confocal microscopy were analysed using Volocity® software. Areas of interest were drawn around the borders of the section (ii) or the B cell follicle based on B220 expression (GREEN) (iv), which allowed the calculation of the respective surfaces. The number of transgenic T cells was calculated based on the intensity of the KJ1.26 staining (RED). (iii and v) Objects smaller than 30 µm and larger than 300 µm were excluded. B) The proportion of transgenic T cells that reside in the follicle was normalized to the number of KJ1.26^+^cells in the section and the surface of the section and follicle. Microscope: Carl Zeiss LSM510 META Confocal, Objectives: Zeiss PH 10×/0.3NA air objective. Images were acquired using Zeiss LSM510 operating software and off-line image analysis (contrast enhancement and noise removal) was performed using Volocity® software. Three random sections per animal were used.
(TIF)

Figure S2 **Phenotype of transferred T and B cells.** Representative plots of intracellular staining for IL-17, IFNγ (A) and IL-21 (B) of polarised Th1 or Th17 DO11.10 cells that were used for adoptive transfers was performed before transfer into IgH^b^ mice. B) The proportion of transgenic B cells that recognise HEL was assessed by their ability to bind biotinylated-HEL. Biotinylated-BSA was used as a negative control.
(TIF)

Figure S3 **Ability of transferred T cells to support cognate B cell expansion and germinal centre formation.** A) Example plots demonstrating identification of transgenic B cells by flow cytometry in the dLNs seven days post-immunisation. Lymphocytes were identified based on the FSC and SSC and transgenic B cells were identified as lymphocytes co-expressing B220 and IgM^a^. B) Example flow cytometry plots of GC B cell staining in the dLNs 3,7 and 10 days post immunisation.
(TIF)

Figure S4 **Expansion of transgenic T cell in spleen and phenotype of transferred population in dLNs.** A and B) Collective data demonstrating the percentage (A) and number of (B) of transgenic T cells at days 3, 7 and 10 post-immunisation amongst spleen cells was assessed by flow cytometry, based on the expression of CD4 and the clonotypic TcR recognised by the KJ1.26 antibody. The grey line represents Th17 immunised recipients, grey dotted line unimmunised Th17 recipients, black line Th1 immunised recipients and black dotted line Th1 unimmunised recipients. C) Representative flow cytometry data demonstrating expression of T-bet (top panel) or RORγt (lower panel) by the transgenic T cells 7 days post-immunisation. Data represent mean ±SEM.*p<0.05, **p<0.01, ***p<0.001 (n = 3).
(TIF)

Figure S5 **Ability of Th1 and Th17 polarised population to proliferate after in-vitro re-stimulation.** MACS sorted CD4^+^T cells from DO11.10 mice were first polarised towards a Th1 or Th17 phenotype, rested for 24 hrs and labelled with CFSE. Cells were restimulated with OVA_323–339_ in the presence of mitomycin C treated splenocytes for 48 hrs and their relative ability to proliferate was assessed by analysis of CFSE dilution. The figure demonstrates a representative flow cytometry plot of CFSE staining of transgenic T cells.
(TIF)

Figure S6 **Expression of TFH markers by the transferred T cell populations.** A) Example flow cytometry plots of PD-1 and CXCR5 expression by CD4^+^KJ1.26^+^transgenic T cells from dLNs 7 days post immunisation. B) Example flow cytometry plots of Bcl-6 levels on CD4+KJ1.26+transgenic T cells from dLNs 7 days post immunisation. In this figure collective flow cytometry data of PD-1^+^CXCR5^high^ (C) and Bcl-6^+^transgenic T cells are also demonstrated. Data represent mean ±SEM.*p<0.05, **p<0.01, ***p<0.001 (n = 4).
(TIF)
