# Supplementary figures and images for: Th17 Effector Cells Support B Cell Responses Outside of Germinal Centres
Source: PLoS One. 2012 Nov 16;7(11):e49715. doi: 10.1371/journal.pone.0049715 (PMC3500323; doi:10.1371/journal.pone.0049715)

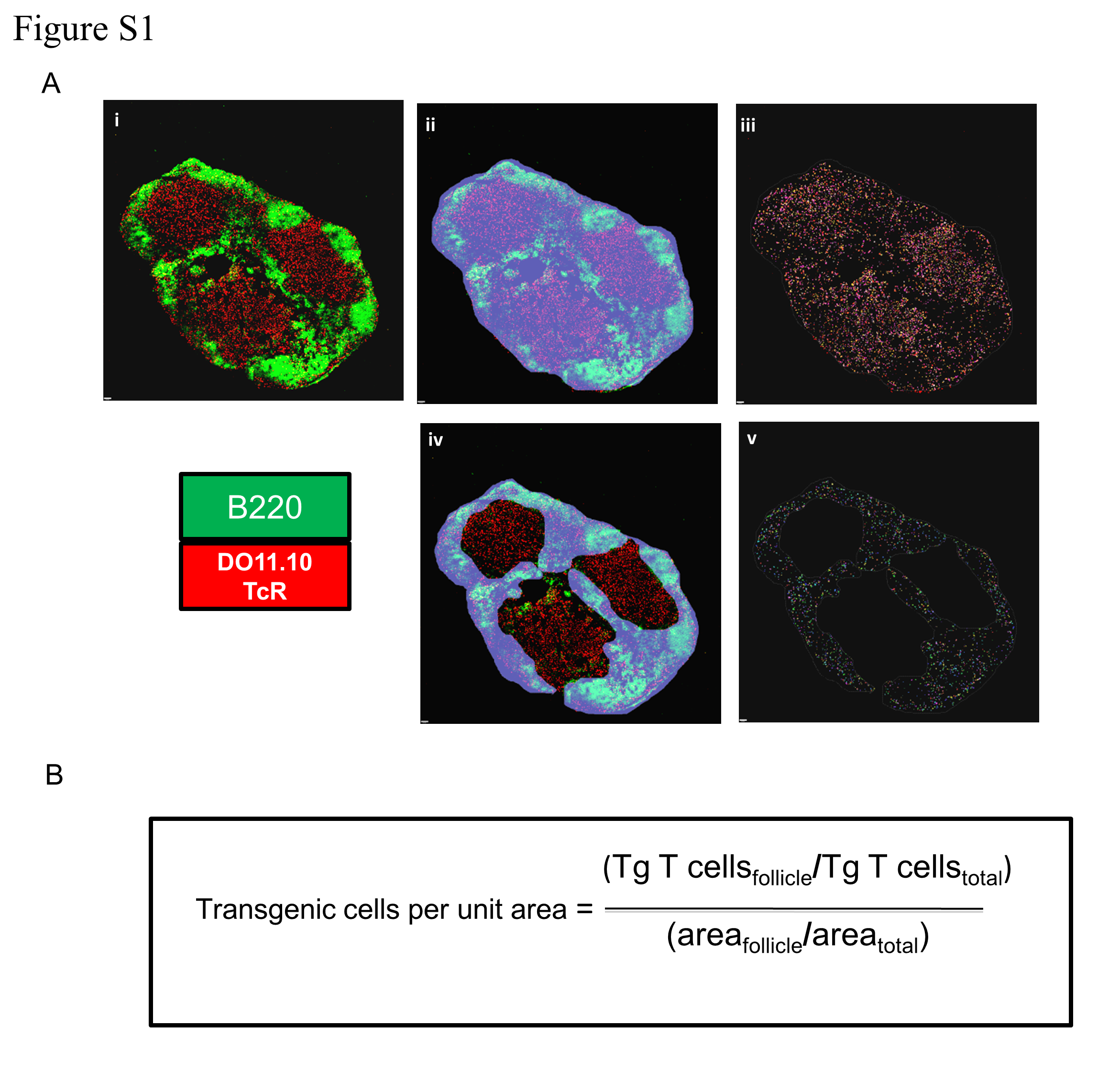

Supplement: Figure S1 — Analysis of localisation of transgenic T cells in the dLNs. A) Tile scan images of dLN sections acquired by confocal microscopy were analysed using Volocity® software. Areas of interest were drawn around the borders of the section (ii) or the B cell follicle based on B220 expression (GREEN) (iv), which allowed the calculation of the respective surfaces. The number of transgenic T cells was calculated based on the intensity of the KJ1.26 staining (RED). (iii and v) Objects smaller than 30 µm and larger than 300 µm were excluded. B) The proportion of transgenic T cells that reside in the follicle was normalized to the number of KJ1.26+cells in the section and the surface of the section and follicle. Microscope: Carl Zeiss LSM510 META Confocal, Objectives: Zeiss PH 10×/0.3NA air objective. Images were acquired using Zeiss LSM510 operating software and off-line image analysis (contrast enhancement and noise removal) was performed using Volocity® software. Three random sections per animal were used. (TIF) [file pone.0049715.s001.tif]

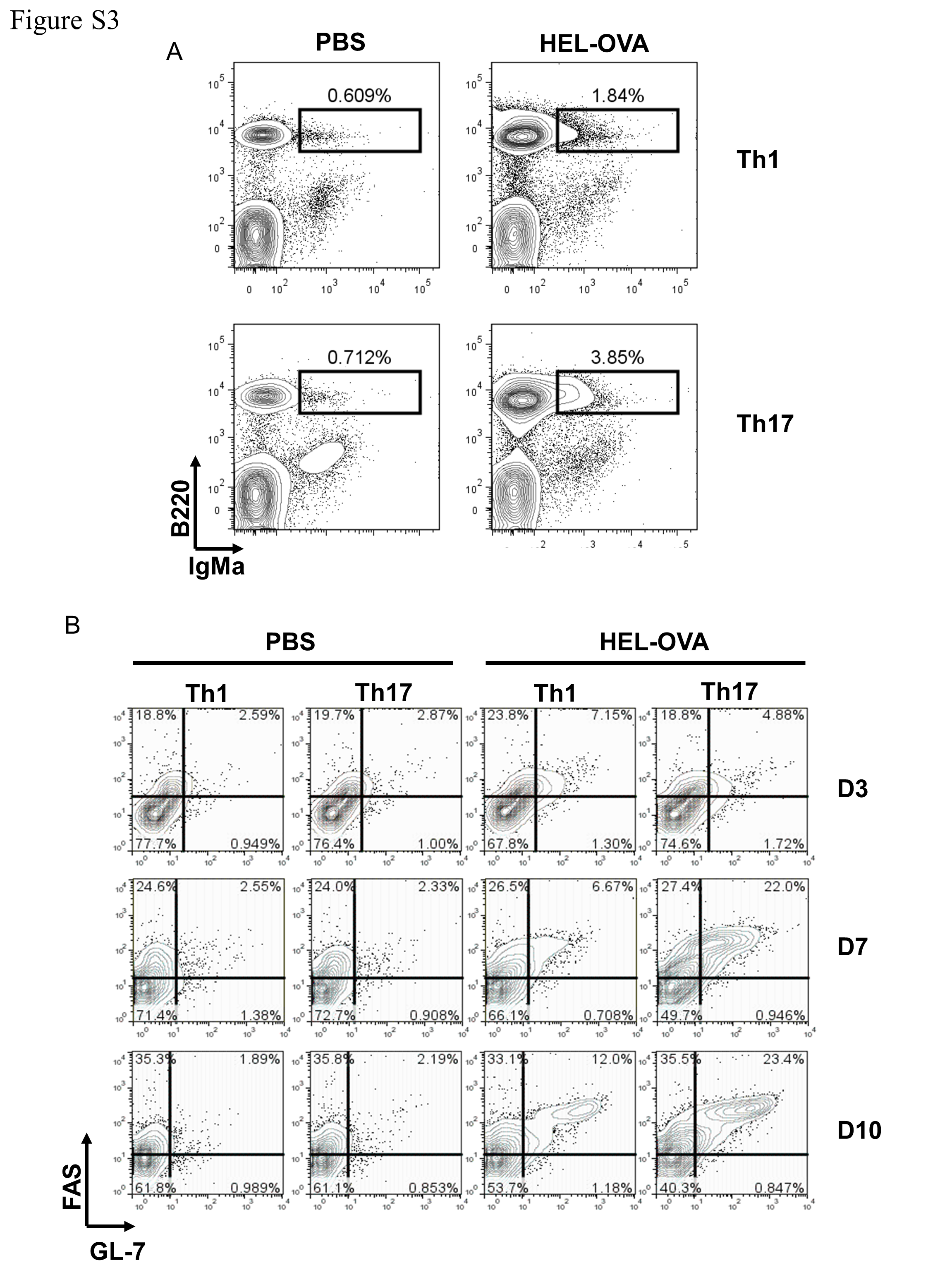

Supplement: Figure S3 — Ability of transferred T cells to support cognate B cell expansion and germinal centre formation. A) Example plots demonstrating identification of transgenic B cells by flow cytometry in the dLNs seven days post-immunisation. Lymphocytes were identified based on the FSC and SSC and transgenic B cells were identified as lymphocytes co-expressing B220 and IgMa. B) Example flow cytometry plots of GC B cell staining in the dLNs 3,7 and 10 days post immunisation. (TIF) [file pone.0049715.s003.tif]

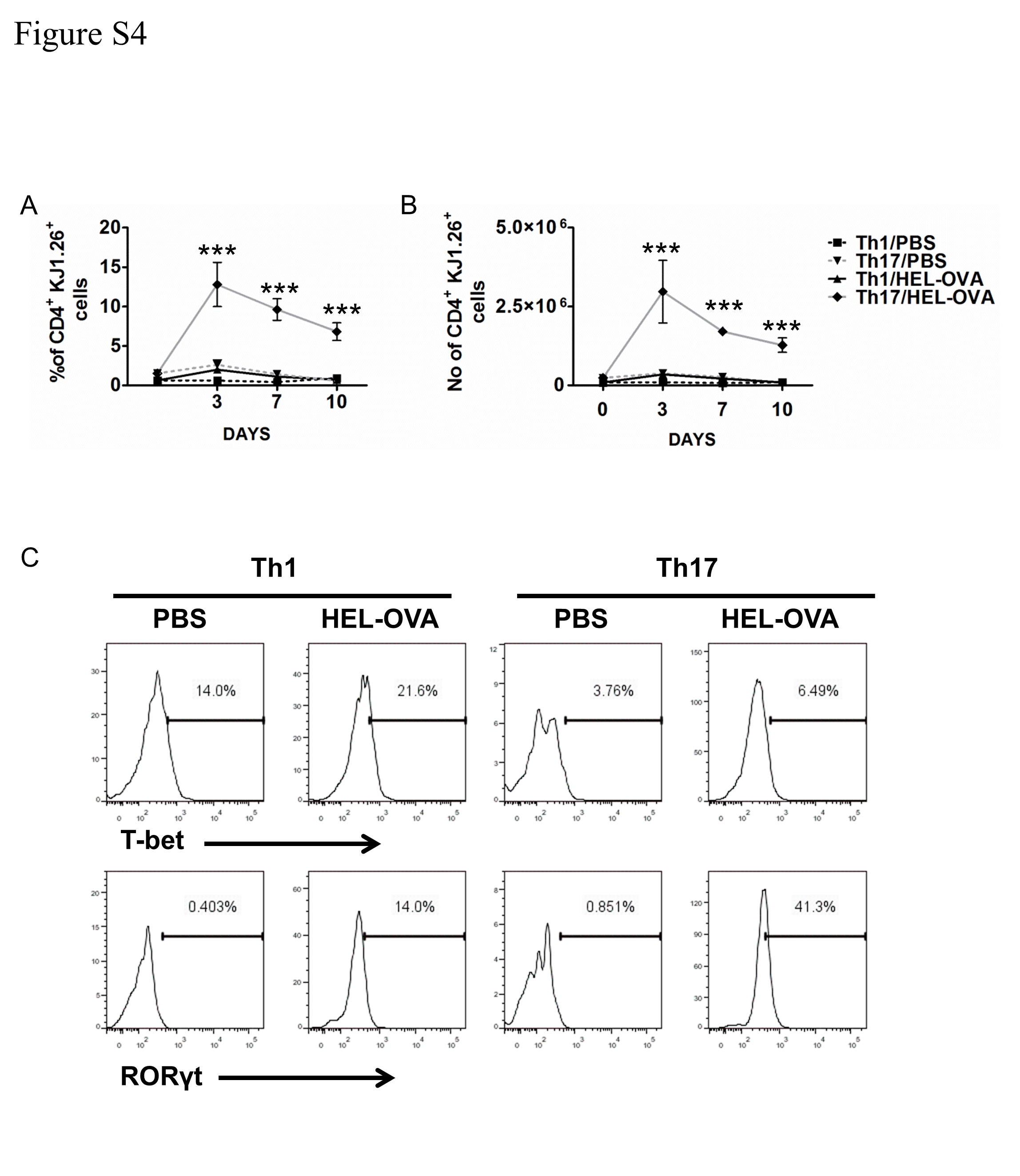

Supplement: Figure S4 — Expansion of transgenic T cell in spleen and phenotype of transferred population in dLNs. A and B) Collective data demonstrating the percentage (A) and number of (B) of transgenic T cells at days 3, 7 and 10 post-immunisation amongst spleen cells was assessed by flow cytometry, based on the expression of CD4 and the clonotypic TcR recognised by the KJ1.26 antibody. The grey line represents Th17 immunised recipients, grey dotted line unimmunised Th17 recipients, black line Th1 immunised recipients and black dotted line Th1 unimmunised recipients. C) Representative flow cytometry data demonstrating expression of T-bet (top panel) or RORγt (lower panel) by the transgenic T cells 7 days post-immunisation. Data represent mean ±SEM.*p<0.05, **p<0.01, ***p<0.001 (n = 3). (TIF) [file pone.0049715.s004.tif]

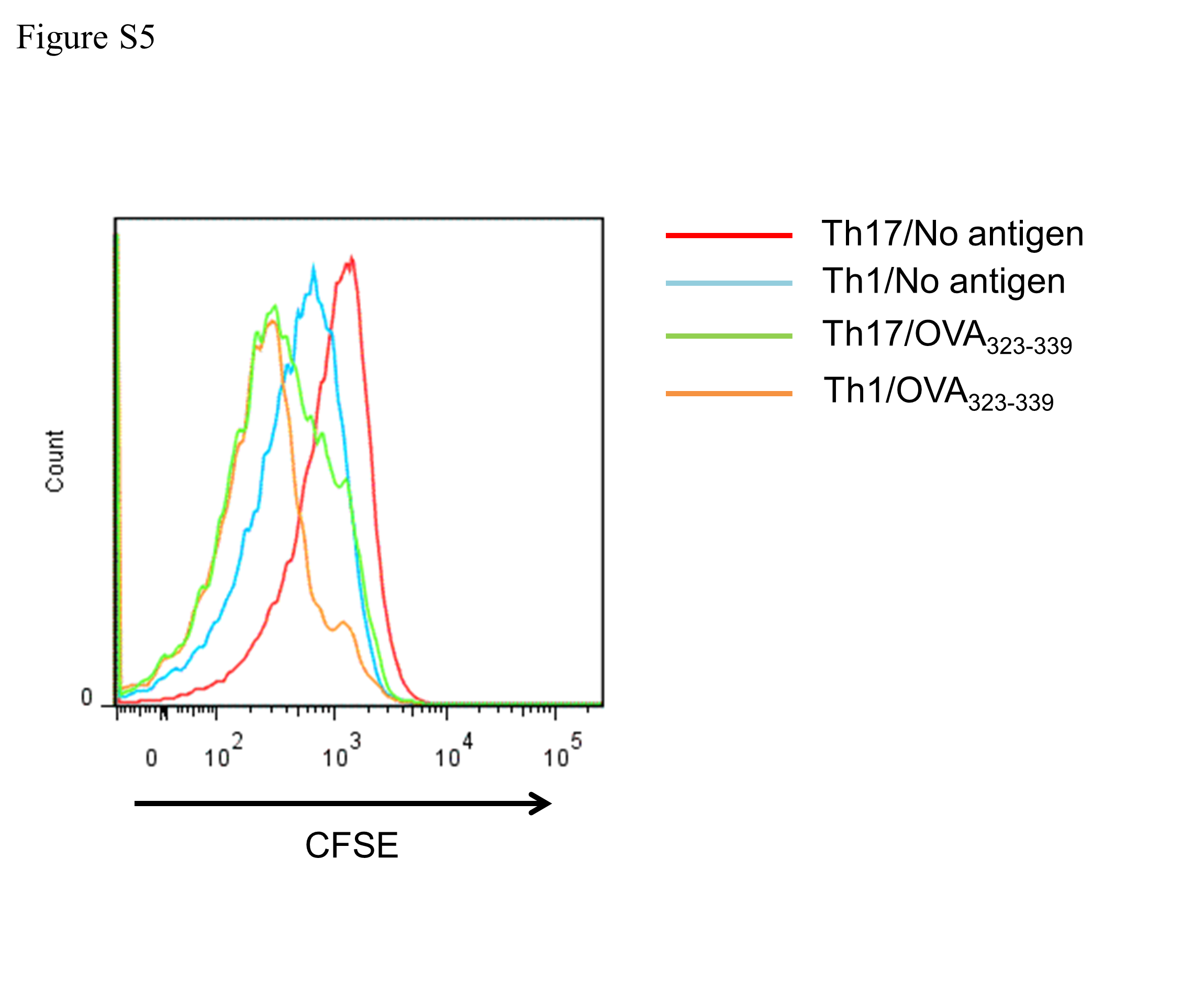

Supplement: Figure S5 — Ability of Th1 and Th17 polarised population to proliferate after in-vitro re-stimulation. MACS sorted CD4+T cells from DO11.10 mice were first polarised towards a Th1 or Th17 phenotype, rested for 24 hrs and labelled with CFSE. Cells were restimulated with OVA323–339 in the presence of mitomycin C treated splenocytes for 48 hrs and their relative ability to proliferate was assessed by analysis of CFSE dilution. The figure demonstrates a representative flow cytometry plot of CFSE staining of transgenic T cells. (TIF) [file pone.0049715.s005.tif]

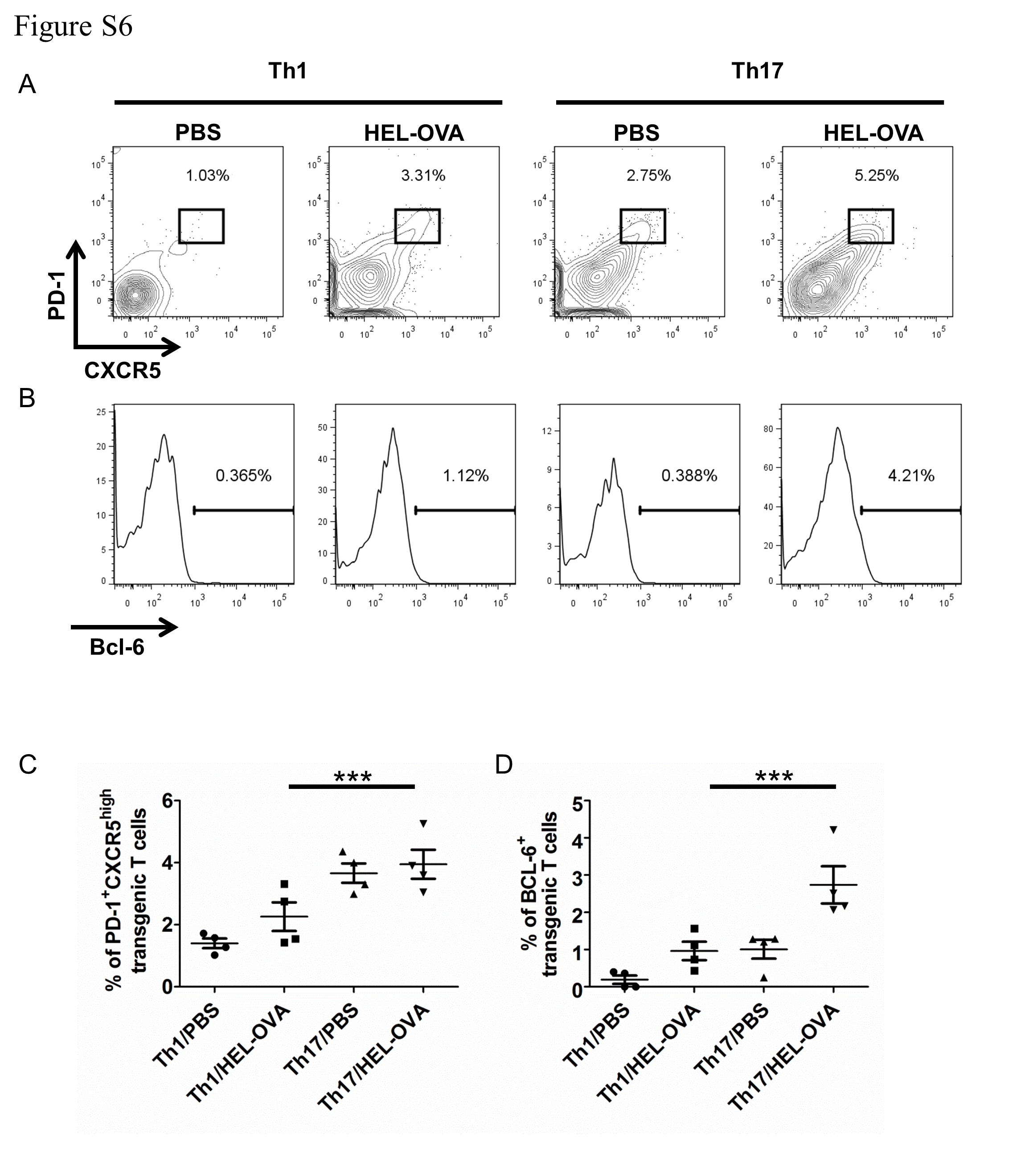

Supplement: Figure S6 — Expression of TFH markers by the transferred T cell populations. A) Example flow cytometry plots of PD-1 and CXCR5 expression by CD4+KJ1.26+transgenic T cells from dLNs 7 days post immunisation. B) Example flow cytometry plots of Bcl-6 levels on CD4+KJ1.26+transgenic T cells from dLNs 7 days post immunisation. In this figure collective flow cytometry data of PD-1+CXCR5high (C) and Bcl-6+transgenic T cells are also demonstrated. Data represent mean ±SEM.*p<0.05, **p<0.01, ***p<0.001 (n = 4). (TIF) [file pone.0049715.s006.tif]
